# A novel “lateral approach short axis in-plane” technique vs. conventional “short-axis out-of-plane approach” for ultrasound-guided internal jugular vein access: a prospective randomized non-inferiority trial

**DOI:** 10.1186/s13089-025-00405-9

**Published:** 2025-01-16

**Authors:** Michal Kalina, Patricia Vargová, Adéla Bubeníková, Roman Škulec, Vladimír Černý, David Astapenko

**Affiliations:** 1 Department of Anaesthesiology, Perioperative Medicine and Intensive Care, Masaryk Hospital in Usti Nad Labem, J. E. Purkinje University, Socialni Pece 3316/12A, 401 13 Usti Nad Labem, Czech Republic; 2 Department of Anaesthesia and Intensive Care Medicine, 3rd Faculty of Medicine Prague, Charles University, Ruska 97, Prague, 100 00 Czech Republic; 3 Faculty of Medicine in Hradec Kralove, Charles University, Simkova 870, Hradec Kralove, 500 03 Czech Republic; 4 Department of Neurosurgery, 2nd Medical Faculty, Charles University and Motol University Hospital, V Uvalu 84, Prague, 150 06 Czech Republic; 5 Department of Anaesthesiology, Perioperative and Intensive Care, Nemocnica Bory, a.S, Ivana Kadlecika 2, Bratislava, 841 03 Slovak Republic; 6 Emergency Medicine Department, Nemocnica Bory, a.S, Bratislava, Slovak Republic; 7 Department of Anaesthesia, Pain Management and Perioperative Medicine, Dalhousie University, Halifax, NS B3H 4R2 Canada; 8 Department of Anaesthesiology and Resuscitation Care, Decin Hospital, U Nemocnice 1, Decin, 405 02 Czech Republic; 9 Department of Anaesthesiology, University Hospital Bulovka, Budinova 2, Prague, 180 00 Czech Republic; 10 Department of Anesthesiology and Intensive Care Medicine, University Hospital Hradec Kralove, Sokolská 581, Hradec Kralove, 500 05 Czech Republic; 11 Faculty of Health Studies, Technical University in Liberec, Studentska 1402, Liberec, 460 01 Czech Republic

**Keywords:** Cannulation, Jugular vein, Centrally inserted venous catheter, Ultrasound, Point of care ultrasound

## Abstract

**Background:**

The cannulation of the internal jugular vein (IJV) is a frequent procedure in critically ill patients. According to the guidelines, real-time ultrasound navigation is recommended. Traditional techniques pose several disadvantages, such as suboptimal needle visualization. Therefore, this non-inferiority trial aimed to describe the novel approach and compare the novel lateral in-plane short-axis approach for IJV access with the conventional short-axis out-of-plane approach.

**Objectives:**

The primary objective of the trial was to prove that the first attempt success rate in the novel technique is non-inferior to the conventional technique. The secondary objectives were to demonstrate that the complication rate and the functional duration of the catheter in the novel technique are not inferior to those in the conventional technique.

**Methods:**

Patients eligible for IJV cannulation were randomly assigned to either the novel technique (Group A) or the conventional one (Group B). The procedure duration, success rate and the number of attempts required were documented. The functionality of the catheter and complications were monitored from insertion until the catheter removal. Standard descriptive statistical methods were employed for the analysis.

**Results:**

A total of 200 subjects were equally divided between Group A and Group B. For the primary outcome, there was no significant difference in first attempt success rate (Group A: 79, Group B: 77, *p* = 0.434). Secondary outcomes, including complications and catheter functional time, did not differ significantly between the groups. However, the novel technique demonstrated a significantly faster procedure time (Group A: 315 s, Group B: 330 s, *p* = 0.016). Notably, the novel approach was linked with significantly larger IJV diameter measured during the procedure (Group A: 18.2 mm, Group B: 12.1 mm, *p* < 0.001).

**Conclusion:**

The novel lateral in-plane short-axis approach for IJV cannulation is a non-inferior alternative with a lower incidence of posterior vessel wall puncture compared to the conventional approach.

## Background

Central venous cannulation is a routine procedure in intensive and perioperative care and constitutes a fundamental skill for physicians. From the beginning, the blind technique based on anatomical landmarks was used [5]. The use of real-time ultrasound navigation for IJV catheterization improved the first attempt success rate and safety of the procedure [7, 8]. Consequently, in the last 30 years, the ultrasound-guided technique has become a standard method [5, 8].

Among the cannulation sites, the internal jugular vein (IJV) stands out as one such location. The IJV can be visualized in a short axis, oblique axis, or long axis ultrasound view. Depending on the puncture technique needle can be visualized out-of-plane or in-plane [2, 6, 8].

Traditionally, IJV is displayed on a short axis with an out-of-plane needle visualization. This method offers a clear view of the jugular vein and adjacent anatomical structures, the carotid artery most prominently. However, the disadvantage is displaying only the needle tip [2, 4, 9] and having the exit site in the red zone of the neck [1]. On the contrary, the long-axis IJV view with an in-plane needle puncture technique (the needle is inserted above the ultrasound probe) provides excellent needle imaging but poses technical challenges for vein visualization and drives the exit site also into the red zone [1]. Moreover, it lacks a view of the carotid artery and surrounding anatomical structures, making it associated with a lower success rate on the first attempt and requiring greater technical expertise from operators [4]. Notably, catheter fixation using this method can be uncomfortable for patients due to the placement in the upper neck. Consequently, this technique is infrequently employed in contemporary medical practice [13].

To overcome the disadvantages that both mentioned techniques pose, we designed a novel cannulation technique. The novel technique displays the IJV on a short axis, and the needle is visualized in-plane. This method combines the benefits of both mentioned techniques. Affording superior needle visualization while concurrently ensuring a comprehensive view of anatomical structures. This non-inferiority trial aimed to describe the novel lateral short axis in-plane puncture technique for IJV cannulation and compare it to the conventional technique in terms of successful first cannulation attempt, time needed to perform the cannulation, complication rate, and functional indwelling time. We hypothesized that there is no lower first-attempt success rate using the novel technique compared to the conventional one.

## Materials and methods

We conducted a prospective randomized non-blinded non-inferiority clinical trial in patients undergoing cannulation of the centrally inserted venous catheter (CICC). The trial was approved by the local ethical committee (Ethics Committee, Masaryk Hospital Usti nad Labem, Czech Republic, reference number 313/3). The trial was conducted in accordance with the Helsinki Declaration and good clinical practice. All patients agreed to be included in the clinical trial and signed an informed consent form. The trial was registered 23rd of May 2022 at, https://clinicaltrials.gov/study/NCT05399108, and assigned by trial registration number NCT0583168.

## Trial design

Patients were randomized into one of the two predefined groups in a 1:1 ratio. In Group A, the novel technique was used for IJV cannulation, whereas, in Group B, cannulation was performed using the conventional technique [13]. The preprocedural evaluation was performed and the diameter of the IJV was measured in the direction of the needle trajectory. A larger IJV was chosen for the cannulation which was performed following the SIC protocol [1]. In both groups, three to five-lumen catheters were used (Teleflex, Wayne, Pennsylvania, USA). The L12-3 linear probe was used with a Fujifilm Sonosite PX ultrasound machine (Bothell, Washington, USA). The time from skin puncture to sterile covering was measured. For the fixation of the catheter StatLock PICC Plus stabilization device (Beckotn, Dickinson and Company, New Jersey, USA) was used. The day after cannulation sterile covering was changed. During this procedure, the distance from the jugulum to the exit site and from the thyroid cartilage to the exit site was measured from the upfront position. There was a daily control of the catheter functionality.

There were four operators involved in the trial. All four operators were skilful in both techniques. Based on the length of the practice of the operators, we assume that each made more than 200 IJV cannulations using the conventional technique. Before the trial started, each operator performed exactly 20 cannulations using the novel technique.

## Population

All patients included in the study were admitted to the general ICU at the Masaryk Hospital in Usti nad Labem, Czech Republic, the tertiary hospital. Eligible participants were enrolled from the 25th of July 2022 till the 30th of January 2024. The CICC placement in both groups was done in the ICU. The inclusion criteria were an adult patient indicated for CICC insertion and a signed informed consent form. The exclusion criteria were as follows: unconsciousness, age <18 years, patient primary indicated for CICC placement into subclavian vein, contraindication of an ultrasound examination (known hypersensitivity to ultrasound gel), contraindication of IJV cannulation (acute skin lesion, phlebitis, neck phlegmon, burns, frostbite, eczema, trauma, thrombosis), anticoagulant therapy, antiaggregant therapy, and the patient’s refusal.

## Methods of cannulation

**Fig. 1:**
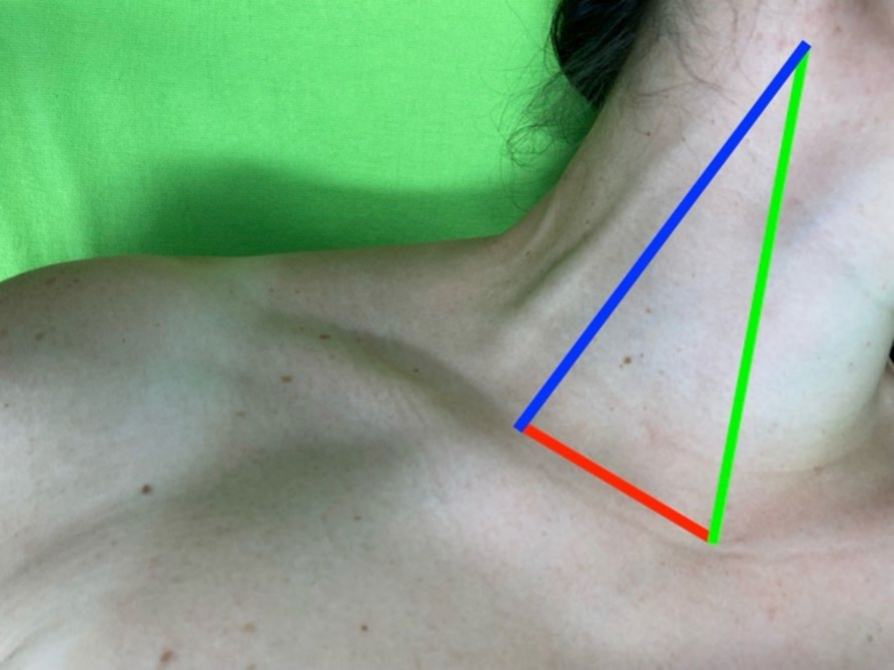
The triangular neck region, clavicle forming base (red), and sternocleidomastoid muscle heads forming sides of the triangle (sternal head green, clavicular head blue)

In group A, a novel cannulation technique was utilized. The technique begins with positioning the patient in a head-down posture to enhance distension of the IJV. A triangular region on the neck is identified, bordered by the clavicle and the sternal and clavicular heads of the sternocleidomastoid muscle (Fig. 1).

**Fig. 2:**
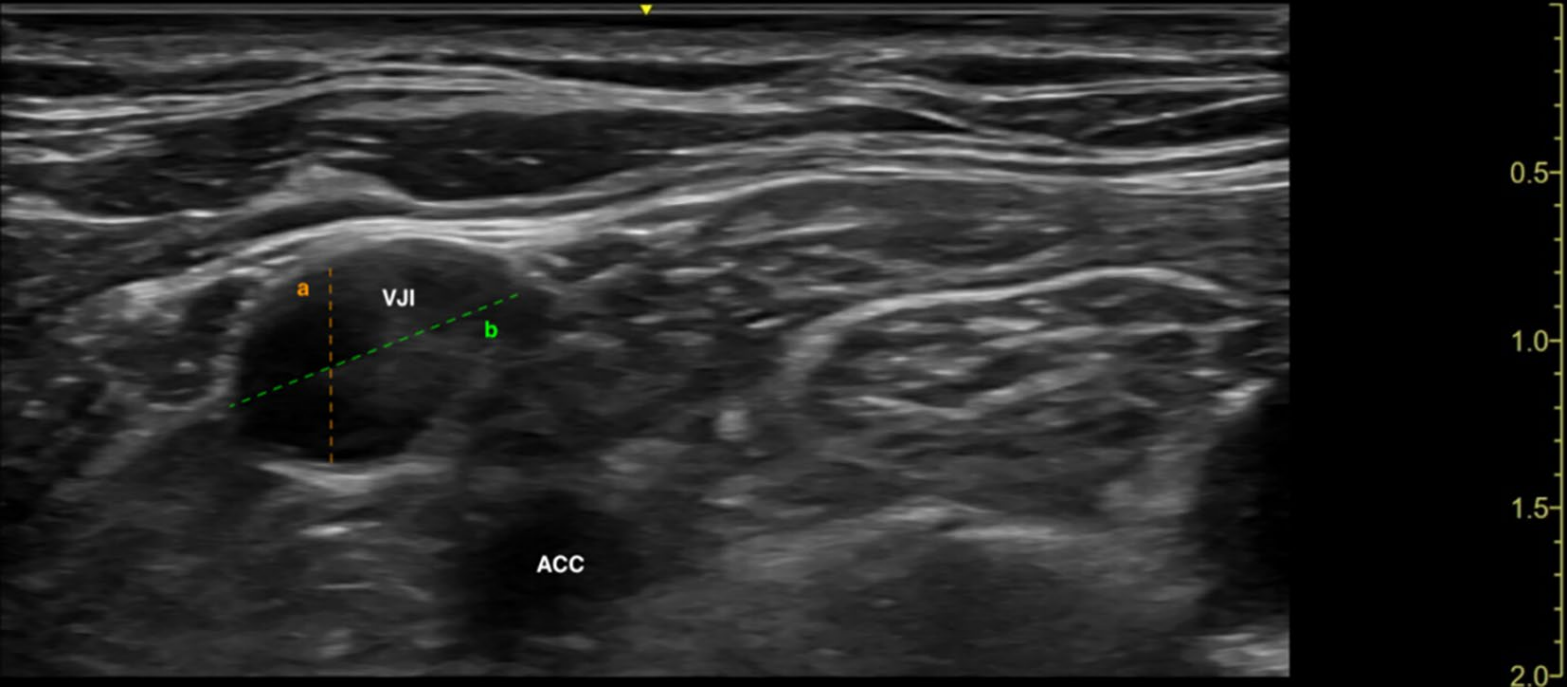
An ultrasound image of the vessel’s short axis (transverse plane). Line A is the diameter of the vessel for the conventional technique, and line B is the diameter for the novel technique. IJV vena jugularis interna, ACC arteria carotis communis

**Fig. 3:**
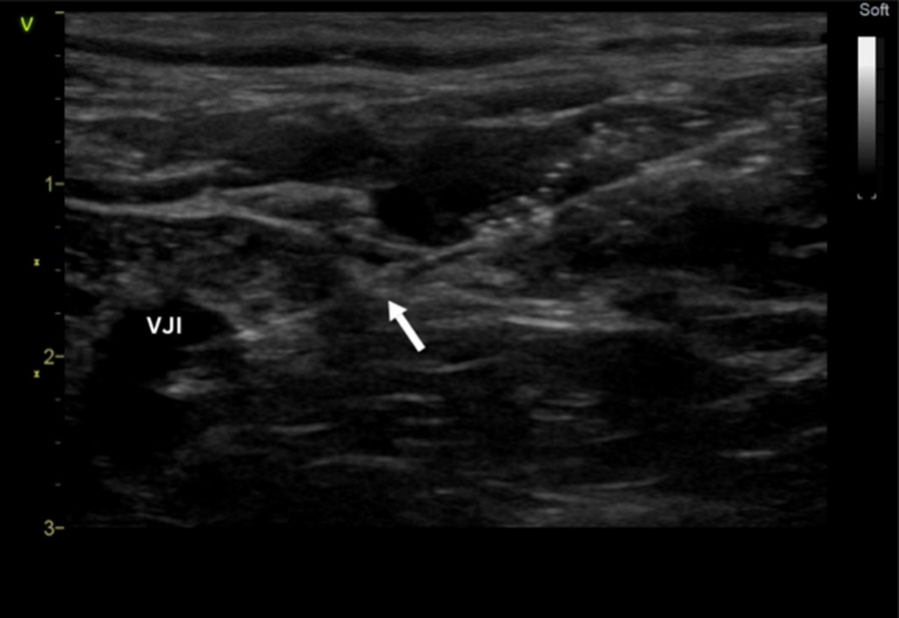
Ultrasound image visualizing needle (white arrow) in-plane as hyperechogenic line entering an IJV

After draping the patient in a sterile manner, ultrasound gel is applied to the base of the triangle, and a high-frequency linear probe is positioned just above the base in a horizontal position. The IJV and carotid artery are visualized (Fig. 2). The introducer needle is inserted lateral to the probe. The needle is advanced towards the IJV, with its trajectory monitored in a long axis on the ultrasound screen (Fig. 3).

**Fig. 4:**
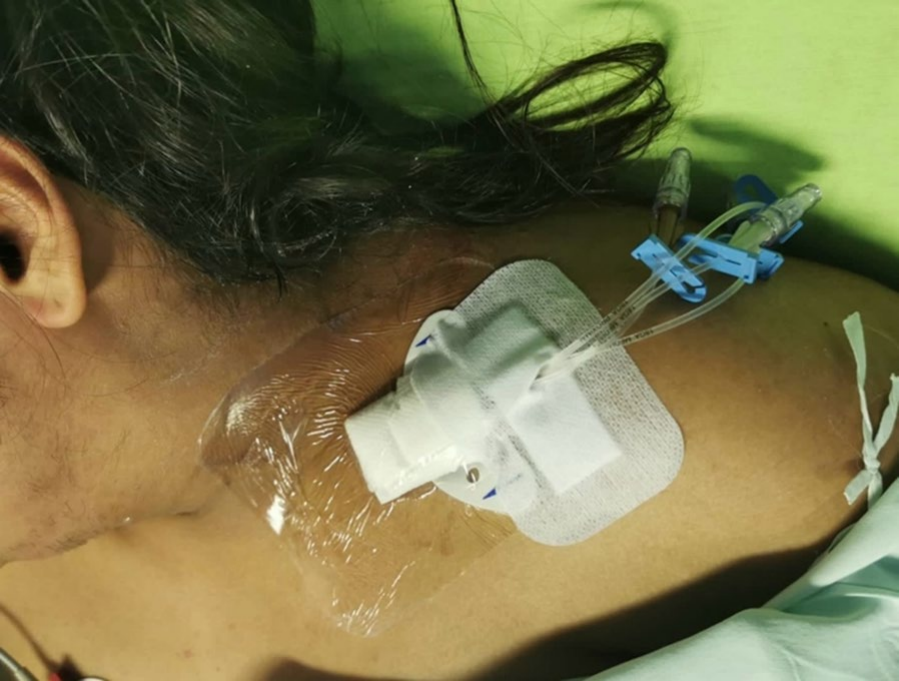
Catheter fixation using the stabilization device in the novel technique in the supraclavicular region

Once the needle successfully enters the IJV, the ultrasound probe is removed, and the standard Seldinger technique is continued to put the catheter in place. Adhesive fixation is then applied to ensure stability, and a sterile covering is placed to conclude the procedure (Fig. 4).

In group B the conventional technique was used [13].

## Data recording and measurement definitions

### Diameter of the vessel

The diameter of the vessel was measured using the caliper function of the ultrasound. The diameter was measured in the direction of the introducer needle trajectory according to the patients’ Group.

### Procedure time

The procedure time was measured from the skin puncture of the patient to the sterile covering of the catheter. The time was measured by the Decathlon Kalenji ONstart 110 stopwatch (Villeneuve d’Ascq, France).

### Periprocedural complication rate

The number of periprocedural complications was measured from the puncture of the skin with the introducer needle upon the extraction of the catheter. The periprocedural complication was defined as follows: carotid artery puncture, hematoma visible on ultrasound, hematoma visible on the skin, thrombosis after catheter placement, neurological impairment of the upper limb on the catheterization site, pneumothorax, chylothorax, guiding wire deformation during catheterization, catheter displacement.

### Distance of the exit site from the jugulum

The distance of the exit site of the catheter from the jugulum was measured the day after cannulation when the sterile covering was changed. The distance was measured with the calibrated meter for PICC length measurement (Teleflex, Wayne, Pennsylvania, USA).

### Distance of the exit site from the thyroid cartilage

The distance of the exit site of the catheter from the thyroid cartilage was measured the day after cannulation when the sterile covering was changed. The distance was measured with the calibrated meter for PICC length measurement (Teleflex, Wayne, Pennsylvania, USA).

### Functional indwelling time of the catheter

The functional indwelling time of the catheter was measured in days from the day of cannulation to the day of the extraction of the catheter. The loss of function was defined as follows: impossible aspiration from one or more lumens, signs of infection, thrombosis related to the catheter, and impossible to flush one or more lumens.

## Objectives

The primary objective of the study was to compare the success rate of the first attempt of the IJV cannulation using the novel technique to the success rate of the first attempt using the conventional technique.

The secondary objectives were to compare the time needed to perform successful cannulation of the IJV using the novel technique and the conventional one, to compare the complication rate of the novel technique and conventional one, and to compare the functional indwelling time of the catheter using the novel technique to the catheter inserted using conventional technique.

## Statistical analysis and randomization

A sample size of 196 subjects was calculated to identify a 20% difference in the primary outcome between the two groups with 90% power with a cut-off for statistical significance *p* = 0.05.

Trial randomization was done through Study Randomizer (2017), a web-based randomization service available at https://www.studyrandomizer.com. A group size was set to 100 participants. Randomization was done by an operator who then performed the cannulation according to the group of participants.

The mean values ± standard deviation (SD) or percentages were calculated as necessary. The normality of the data was evaluated according to the Kolmogorov- Smirnovov test. Differences in categorical variables were standardly evaluated using the chi-square test and Pearson coefficient. Comparisons of two continuous variables were calculated using t-tests for parametric variables or the Mann–Whitney U test for non-parametric variables for independent samples. All calculations were performed in an open-source R environment using the ggplot2 library (v4.1.2, R Core Team (2021). R: A language and environment for statistical computing. R Foundation for Statistical Computing, Vienna, Austria, https://www.R-project.org/).

## Results

**Fig. 5:**
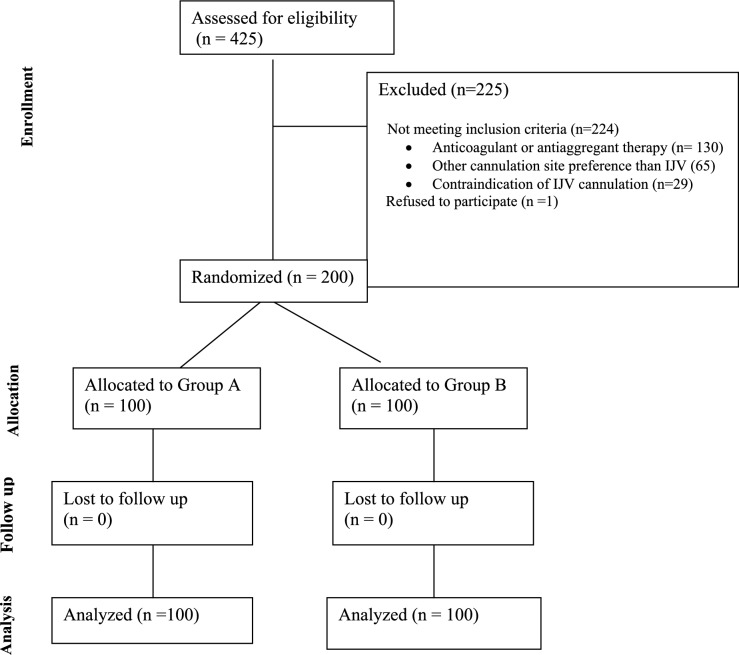
Diagram of the enrolment process

A total of 200 patients met inclusion criteria and were randomized into two groups equally (Group A = 100, Group B = 100; Fig. 5).

We did not find any significant difference regarding demography between the groups. The demography of the study population is presented in detail in Table 1. We did find significant difference in the site of the cannulation with predominance to the left (Group A: right 36, left 64; Group B: right 51 left 48, *p* = 0.027).

**Table 1:** Demography of the study population, data are presented as mean ± standard deviation of percentage as appropriate, *p* values were calculated using chi-squared test for categorical and t-test for continuous variables comparison

	Overall	Group A	Group B	*p*-value
N. of patients	200	100	100	
Age [years]	61.2 ± 15.7	60.2 ± 17.4	62.3 ± 13.8	0.364
Gender [%]				0.570
Females	92 (46)	48	44	
Males	108 (54)	52	56	
Weight [kg]	81.8 ± 21.2	80.9 ± 22.4	82.7 ± 20.0	0.553
Height [cm]	169.6 ± 11.1	169.2 ± 10.9	170.1 ± 11.3	0.546
BMI	28.5 ± 7.5	28.2 ± 7.1	28.9 ± 7.9	0.515
Neck circumference [cm]	42.0 ± 5.9	41.4 ± 6.7	42.7 ± 4.9	0.188
APACHE II	16.1 ± 7.9	15.7 ± 7.1	16.3 ± 8.6	0.623
Mechanical ventilation	77 (38.5)	36	41	0.467

*BMI* body mass index

We did not find a statistically significant difference between the success rate of the first attempt between the groups (Group A 79, Group B 77, *p* = 0.434). However, we find that the time needed for successful cannulation was statistically significantly faster in Group A (Group A 315 s, Group B 330 s, *p* = 0.016). The complication rate (Group A 5, Group B 12, *p* = 0.076) and functional indwelling time (Group A 11.4, Group B 11.5, *p* = 0.914) did not differ significantly between the groups. In detail, it is presented in Table 2. The incidence of different periprocedural complications is presented in detail in Table 3.

**Table 2:** Measurements for main and secondary objectives

	Overall	Group A	Group B	*p*-value
First attempt successful rate	156	79	77	0.434
Cannulation time [s]	327 (261–420)	315 (250–386)	330 (282–458)	0.016
Complication rate	17	5	12	0.076
Functional time [days]	11.4 ± 6.6	11.5 ± 6.7	11.4 ± 6.4	0.914

Cannulation time is presented in seconds with standard maximum and minimum deviation, functional indwelling time of the catheter is presented in days. *P*-value stands for t-test

**Table 3:** Observed different periprocedural complications

Complication	Overall	Group A	Group B	*p*-value
Carotid artery puncture	5	1	4	0.088
Catheter displacement	8	2	6	0.075
Guiding wire deformation	3	1	2	0.157
Hematoma on the skin	1	1	0	0.159

*P*-value stands for t-test

We observed a significantly bigger mean distance from the insertion site of the CICC to the jugulum and thyroid cartilage (Group A 9.7 cm, Group B 7.6 cm, *p* < 0.001 and Group A 9.3 cm, Group B 7.1 cm, *p* < 0.001) even though the mean neck circumference did not differ between the groups (*p* = 0.188) as described in Table 4. We observed a significantly larger diameter of the IJV in Group A compared to Group B (Group A 18.2 mm, Group B 12.1 mm, *p* < 0.001).

**Table 4:** The mean distance from the insertion site and neck circumference measured in cm, *p* values were calculated using a t-test for continuous variable comparison, data are presented as mean ± standard deviation

.	Overall	Group A	Group B	*p*-value
Neck circumference [cm]	42.0 ± 5.9	41.4 ± 6.7	42.7 ± 4.9	0.188
Distance from jugulum to insertion site [cm]	8.7 ± 2.3	9.7 ± 2.1	7.6 ± 1.9	**<0.001**
Distance from insertion site of the CICC to thyroid cartilage [cm]	8.3 ± 2.4	9.3 ± 2.2	7.1 ± 2.1	**<0.001**

*CICC* centrally inserted venous catheter

We did not observe any failed cannulation in either group. There was no significant difference between the groups in the number of needed attempts for successful cannulation, presented in detail in Table 5.

**Table 5:** Total number of needed attempts for successful cannulation in group A and Group B

Number of attempts	Overall	Group A	Group B	*p*-value
1	156	79	77	0.434
2	33	18	15	
3	8	3	5	
4	1	0	1	
6	2	0	2	

*P*-value stands for a t-test

## Discussion

The main findings of the trial are that the lateral short axis in the plane technique is non-inferior to the conventional one regarding the first cannulation attempt success rate, time of the procedure, and complication rate. Another important finding of the trial is that the novel technique is associated with a significantly larger diameter of the IJV and that the insert side is significantly further from the thyroid cartilage and jugulum.

We discovered that the novel technique was statistically significantly faster compared to the conventional technique. However, the difference was only 15 s. The mean duration of the procedure time in our trial was nearly six minutes. In other trials, the reported procedure duration time is about 3 min [11, 12]. However, in these trials, the time is measured from the insertion of the guiding needle till catheter insertion. In our trial, we measured the time until the sterile covering of the catheter to compare the time that is needed for the catheter to be prepared for use. We can conclude that 15 s is a short time compared to the total procedure duration time and therefore there is no clinical relevance in the difference in duration procedure time between the novel technique and conventional one.

We explain the larger diameter of the IJV in the novel technique by the fact that the cannulation site in the novel technique is closer to the heart and therefore the vessel has a bigger diameter. The second reason for this finding could be that the vessel is never a perfect circle even when displayed on a short axis and we measured the diameter in the direction of the needle trajectory. The larger diameter of the IJV was linked also to oblique axis visualization compared to conventional short axis visualization in published trials [3, 4, 13]. Therefore, we can predict that the novel technique described in our trial could be associated with a lower incidence of posterior vessel wall puncture (PVWP) same as the oblique technique compared to the conventional technique [13].

In the last decade, there have been several publications regarding alternative techniques for IJV cannulation to lower the complication rate by using in-plane needle visualization and moving the exit site to the supraclavicular region to reduce infectious complications [2, 6, 9, 13]. In our trial, we described a novel cannulation technique. Our findings concord with other trials that described and investigated oblique axis IJV catheterization which is another technique using in-plane needle visualization. Same as Wilson [13] we did not find significantly more complications in the novel technique compared to the conventional technique. We admit that opposite to Wilson we did not measure the PVWP rate which is one of the most common complications of IJV cannulation [3]. However, in our trial, we measured the incidence of post-cannulation thrombosis that is linked to PVWP [13]. The incidence did not differ between the novel technique and the conventional technique.

Our results regarding complication rate correspond to results presented by Song [10] who described a similar cannulation technique for the insertion of large bore catheters in his trial.

We admit several limitations of the study. All the cannulations were done by operators who are experienced in the conventional technique, oblique technique, and novel technique. Therefore, the trial results cannot be generalized to operators without experience with the cannulation techniques. Because the trial is monocentric the cannulation time and complication rate may be center-specific which is another limitation of the generalisability of the findings. We also did not include paediatric patients. Lastly, we did not measure the infectious complication rate. The reason for not measuring is that the trial is focused on the procedure of the catheter insertion. Infectious complications are largely related to nursing care and proximity to the oropharyngeal secretion. We can assume that the infectious complication rate using a novel technique may be lower as the exit site is moved from the neck (red zone) to the supraclavicular region (yellow zone). This change of exit site is associated with lower infectious complications, as showed by Brescia [1].

In our study, we did not specifically examine the difference in learning curves between the novel and conventional techniques. Currently, there is a lack of relevant data available to determine whether the novel technique offers a faster learning curve. However, it is common practice for physicians undergoing anaesthesia training to initially be trained in regional anaesthesia with in-plane needle visualization. This suggests that there is a possibility that the novel technique may have a faster learning curve than the conventional method. Further research is needed to thoroughly investigate this hypothesis.

We can conclude that the novel technique that we described is not inferior to the conventional technique. We also came to very similar findings as authors describing the oblique axis cannulation technique. Therefore, the novel technique can be another valuable alternative way for IJV cannulation with lower PVWP incidence compared to the conventional technique.

## Conclusion

The novel cannulation technique can be an alternative to the conventional cannulation technique because it is linked with a greater diameter of the vessel during puncture and therefore there is a predicted lower incidence of PVWP.

## Data Availability

The datasets used and/or analysed during the current study are available from the corresponding author on reasonable request.

## Abbreviations

ACC: Commune carotid artery
BMI: Body mass index
CICC: Centrally inserted venous catheter
IJV: Internal jugular vein
SCM: Sternocleidomastoid muscle
SD: Standard deviation
PWP: Posterior vessel wall puncture
